# Lipid Regulators during Atherogenesis: Expression of LXR, PPAR, and SREBP mRNA in the Human Aorta

**DOI:** 10.1371/journal.pone.0063374

**Published:** 2013-05-23

**Authors:** Tatyana A. Shchelkunova, Ivan A. Morozov, Petr M. Rubtsov, Yuri V. Bobryshev, Igor A. Sobenin, Alexander N. Orekhov, Irina V. Andrianova, Alexander N. Smirnov

**Affiliations:** 1 Biological Faculty, Lomonosov Moscow State University, Moscow, Russia; 2 Engelhardt Institute of Molecular Biology, Russian Academy of Sciences, Moscow, Russia; 3 Institute of General Pathology and Pathophysiology, Russian Academy of Medical Sciences, Moscow, Russia; 4 Faculty of Medicine, University of New South Wales and St Vincent’s Hospital Sydney, Kensington, NSW, Australia; 5 School of Medicine; University of Western Sydney, Campbelltown, NSW, Australia; 6 Russian Cardiology Research and Production Complex, Moscow, Russia; 7 Institute for Atherosclerosis Research, Skolkovo Innovation Center, Moscow, Russia; 8 Institute of Biomedical Problems, Russian Academy of Sciences, Moscow, Russia; Brigham and Women’s Hospital, Harvard Medical School, United States of America

## Abstract

Transcription factors LXRs, PPARs, and SREBPs have been implicated in a multitude of physiological and pathological processes including atherogenesis. However, little is known about the regulation of these transcription factors at different stages of atherosclerosis progression. Quantitative real-time polymerase chain reaction (qRT-PCR) was used to compare the contents of mRNAs in pairs intact-injured aorta fragments taken from the same donors. Only minor changes in LXRα, LXRβ, PPARα, PPARγ, SREBP1, and SREBP2 mRNA levels were found in initial lesions as compared with intact non-diseased tissue. The contents of all mRNAs but SREBP2 mRNA were found to be progressively up-regulated in fatty streaks and fibrous lipoid plaques. These changes were only partially reproduced in cultured macrophages upon lipid loading. Wave-shaped changes in abundance of correlations between given group of mRNAs and 28 atherosclerosis-related mRNA species in the course of atherogenesis were observed. The impact of specific mRNA correlations on the total correlations also significantly varied between different lesion types. The study suggests that the extent and forms of LXR/PPAR/SREBP participation in intima functions vary nonlinear in individual fashion in atherogenesis. We speculate that the observed changes in mRNAs expression and coupling reflect shifts in lipid ligands availability and cellular composition in the course of atherosclerosis progression.

## Introduction

Local inflammation and lipid accumulation are two hallmarks of atherosclerosis. These processes are mutually dependent, so that the final outcome, i.e. plaque formation, is the same for both lipid and inflammatory stimuli (reviewed in [1]). The balance between lipids entrance into vessel wall, their local biosynthesis and degradation, as well as reverse transfer is maintained mainly by oxysterol sensors LXRα,β (liver X receptors alpha, beta), fatty acids sensors PPARα,β/δ,γ (peroxisome proliferator-activated receptors alpha, beta/delta, and gamma), and SREBP1,2 (sterol response element-binding proteins 1, 2) whose activity is inhibited by cholesterol at the level of post-translational processing (reviewed in [2]–[7]). All these proteins are transcription factors that interact with response elements found in a multitude of genes engaged in lipid turnover including their own genes. Moreover, LXRs and PPARs were found to participate in negative regulation of pro-inflammatory genes while, in turn, being the objects of inflammatory stimuli (reviewed in [8], [9]). It is not fully understood why the system of lipid homeostasis fails to prevent foam cell formation and following plaque development. As an explanation, inefficient targeting of modified lipoproteins for degradation was proposed, although underlying mechanisms remain uncertain.

The comparison between healthy humans and patients with familial hypercholesterolemia showed that normally, approximately 2/3 of low density lipoprotein (LDL) is eliminated from the bloodstream via LDL receptor-mediated endocytosis (reviewed in [10]). Many risk factors such as dyslipidemia, diabetes mellitus, smoking, etc. provoke enzymatic and non-enzymatic formation of modified LDLs (mLDLs) which enter the cell through various scavenger receptors (such as SR-A and CD36) mediated endocytosis, phagocytosis and by pinocytosis [11]. Within the cell, cholesterol is distributed between lysosomal compartment and lipid droplets as esters with fatty acids formed by acyl-CoA-cholesterol acyltransferase (ACAT). Intracellular transfer is performed using endosomes and autophagosomes. Along with cholesterol import, a system of endogenous cholesterol biogenesis exists. Unlike fatty acids, cholesterol cannot be completely disrupted in the body. Its elimination occurs via bile, in forms of free cholesterol, cholesteryl esters and bile acid derivatives synthesized in the liver. From arterial wall, an excess of cholesterol is removed by a system of reverse transfer that includes liver-derived cholesterol acceptors, ATP-binding cassette transporters, and accessory proteins, such as receptor for high density lipoprotein (HDL), SR-BI, and apolipoprotein E (ApoE) which enhances the capacity of HDL. As a preliminary step, cholesterol esters are hydrolyzed by cholesteryl ester hydrolase (CEH). The function of this cleaning is usually attributed to macrophages, but it is not obvious since, as shown here, all these components of reverse transfer are expressed (at the level of transcription) in resident cells of the aortic intima.

Our current knowledge about artery wall biology is based primary on the studies utilizing macrophages which compose, however, only minor cell population even in advanced atherosclerotic lesions. Moreover, several populations of macrophages with different phenotypes can co-exist within the aorta [12]. Less is known about more numerous resident cells of the vessel including smooth muscle cells which were shown to contribute to foam cell formation (reviewed in [13]). So, it is important to analyze control mechanisms of lipid homeostasis in the majority of arterial cells during the development and progression of atherosclerotic lesions.

## Materials and Methods

This study was kept in accordance with the Helsinki Declaration of 1975 as revised in 1983. It was approved by the local ethics committees of the Russian Cardiology Research and Production Complex, Moscow, and Institute for Atherosclerosis Research, Skolkovo Innovation Center, Moscow, Russia. This study utilized autopsy tissue specimens. Written informed consent for the use of the tissue samples for our study was obtained from relatives of subjects who died from accidental death in each case.

### Artery Samples

This study used aorta samples extracted during autopsies from 13 men aged 31–55 years (median 49 years) and 6 women aged 39–57 years (median 48 years) 4–6 hours after accidental death. The vessels were dissected longitudinally and washed with PBS. Upon macroscopic examination, dissected fragments were divided in half for biochemical and histological analyses. For mRNA measurements, the intima from the apparently normal and the atherosclerotically damaged areas (types I, II, and Va, i.e. initial lesions, fatty streaks, and fibroatheroma or fibrolipid plaques) were mechanically separated, frozen in liquid nitrogen, and kept at −70°C. No lesion shoulders were taken for analysis. The ascribing of samples to certain morphological types of atherosclerotic damages was confirmed microscopically according to American Heart Association classification [14], [15]. Comparisons between intact and injured tissue were performed in pairs of intact/injured fragments taken from the same donors. Such pairs included 17 fragments with type I lesions, 18 fragments with type II lesions, and 11 fragments with type Va lesions.

### Isolation of Monocyte-derived Macrophages

Mononuclear cells were isolated from the fasting venous blood of 8 healthy volunteers (five men and three women) aged 21–57 years with their written informed consent. The isolation was performed by centrifugation on a Ficoll-Paque Plus (Amersham Biosciences; Uppsala, Sweden) density gradient according the manufacturer’s protocol.

To prepare the monocyte-derived macrophages, mononuclear cell suspensions in DMEM (GibcoBRL Life Technologies; Grand Island, NY, USA) containing 10% fetal bovine serum (ICN Biochemicals Inc.; Costa Mesa, CA, USA), 2 mM glutamine, 100 mg/mL kanamycin and 2.5 mg/mL fungizone were plated onto 35-mm plastic culture dishes (Corning Costar; Corning, NY, USA) at a density of 3×10^6^ cells per dish. Cells were cultivated at 100% humidity and 37°C in a CO_2_ incubator (5% CO_2_ and 95% atmosphere air) for 7–10 days with medium changes every other day.

### Macrophage Loading with Lipids

Lipid-loaded monocyte-derived macrophages were prepared using freshly isolated naturally modified LDLs which were isolated from the pooled serum of patients with diagnosed atherosclerosis (with their written informed consent) by a two-stage ultracentrifugation in a discontinuous density gradient of sodium bromide. The LDL fraction thus obtained was dialyzed against PBS for 12 hours at 4°C and sterilized by filtration using a pore size of 0.45 µm. A volume of 100 µg/mL of mLDL was added to the cells and incubated for 18 hours. Control cells were incubated without added LDLs. As an additional control, LDLs from pooled serum of healthy blood donors were used instead of mLDLs. Cells were then washed three times with PBS, lysed with trizol, and kept at −70°C or used for cholesterol measurements.

To confirm mLDL-induced cholesterol accumulation in the macrophages, cells were extracted three times with a hexan/isopropanol mixture (3:2, v/v). The cholesterol content was determined enzymatically using a reagents kit from Biocon® Diagnostik (Marienhagen, Germany). The protein content was measured with Folin reagent [16].The intracellular cholesterol level was normalized to the protein content in each sample. As a result, 2 to 3-fold increase in cholesterol content was observed upon incubation of macrophages with mLDLs. No cholesterol accumulation was found with native LDLs of healthy donors.

### RNA Isolation and Real-time PCR

RNA was isolated by a homogenization of the frozen samples taken from the vessel walls in TRIzol Reagent (Invitrogen; Carlsbad, CA, USA). The RNA was then treated with chloroform, precipitated with isopropanol, and washed with ethanol. The synthesis of cDNA was performed on 2–6 µg of total RNA using a Promega ImProm_II™ Reverse Transcription System kit (Promega Corporation; Madison, WI, USA). The synthesized cDNA was used as a template for quantitative real-time polymerase chain reaction (qRT-PCR) on a Rotor-Gene 3000 amplifier (Corbett Research; Sydney, Australia) with a kit of reagents including the intercalating dye SYBR Green I (Syntol; Moscow, Russia) as recommended by the manufacturer. The details of amplification were described earlier [17]. The primers for the most part of mRNAs were chosen using the Beacon Designer 6.00 program (www.PremierBiosoft.com) and described previously [18]. The primers for the second house-keeping gene, guanine nucleotide-binding protein, beta-peptide 2-like 1 (GNB2L1) were the same as in report of Ishii et al [19]. To check for the absence of products amplified from genomic DNA, isolated RNA was used as a template. Amplified products were sequenced using an ABI PRISM® BigDye™ Terminator v.3.1 kit of reagents and an ABI PRISM 3100-Avant automated DNA sequencer to confirm the expected sequences available in relevant databases. No new DNA sequences were revealed in this study. The results were included only when the melting temperature and the electrophoretic mobility of the amplified products corresponded to the expected values. DNA sequencing was performed in the Center of Collective Use “Genome” at the Engelhardt Institute of Molecular Biology. The content of the particular mRNAs was normalized to glyceraldehyde-3-phosphate dehydrogenase (GAPDH) mRNA content as an internal reference control and expressed as percent.

### Immunohistochemistry

Tissue blocks of aortic wall segments were cross-cut into 6 µm tissue sections. For immunostaining, after elimination of endogenous peroxidase activity by 3% H_2_O_2_, sections were preincubated with normal non-immune serum and then tested by avidin-biotin complex (ABC) using a standard ABC immunoperoxidase method. LXRα, PPARγ and SREBP1 were identified using antibodies from Santa Cruz Biotechnology Inc. (LXRα Antibody (P-20): sc-1202; dilution: 1:200; PPARγ Antibody (B-5): sc-271392; dilution: 1:200; SREBP-1 Antibody (C-20): sc-366; dilution: 1:200; accordingly). After washing in tris-phosphate buffered saline (TPBS), pH 7.6, the sections were incubated with an appropriate biotin-labelled secondary antibody, followed by a treatment with avidin-biotin complex (Elite ABC, Vector PK61000). After washing in TPBS, brown staining was produced by 5 min treatment with 3,3′-diaminobenzidine (DAB). All the incubations were completed at room temperature. For negative controls, the first antibodies were omitted or the sections were treated with an immunoglobulin fraction of non-immune serum as a substitute for the primary antibody. None of the negative control sections showed positive immune staining. Counterstaining was performed with Mayer’s haematoxylin.

### Statistical Analysis

The results were analyzed with the Statistica 7.0 program. Independent samples were compared using the Mann-Whitney U-test. Correlations were evaluated using the Spearman rank test. Differences or correlations were considered significant at *p*<0.05. Mean ± SD values are shown. Correlation graph weight values G_h_ were calculated as sum of modules of statistically significant pairwise correlation coefficients (*r_kl_*): G_h_ = ∑ |*r_kl_*| [20].

## Results

### The Contents of mRNAs in Intact Aorta and Aorta Fragments with Atherosclerotic Injures

To validate the relevancy of GAPDH mRNA as reference point, we have analyzed the expression of the additional housekeeping gene, GNB2L1, in intact and injured aorta fragments from several donors. The ratios of GNB2L1 mRNA contents in injures to its contents in intact aorta fragments, as normalized by GAPDH mRNA, were close to 1.0 for lesions of type I, II, and Va (0.94±0.38 (n = 6), 0.96±0.24 (n = 6), and 0.88±0.11 (n = 4), respectively). The data suggests that the expression of two housekeeping genes does not vary significantly during atherogenesis.

**Figure 1:**
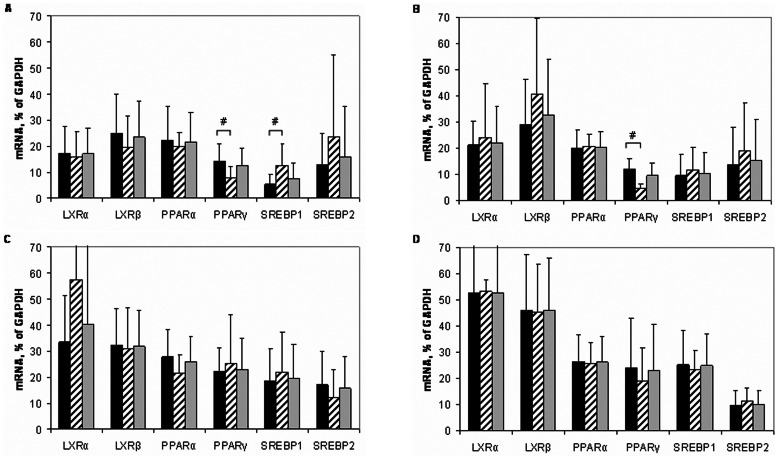
Expression levels of mRNAs encoding for lipid sensors/transcriptional regulators in aortas from men (black bars), women (hatched bars), and in combined M+F population (grey bars). Symbol # makes a note gender difference. A, intact aortas (men, n = 15, women, n = 6); B, lesions of type I (men, n = 11, women, n = 6), C, lesions of type II (men, n = 13, women, n = 5), D, lesions of type Va (men, n = 9, women, n = 2).

**Figure 2:**
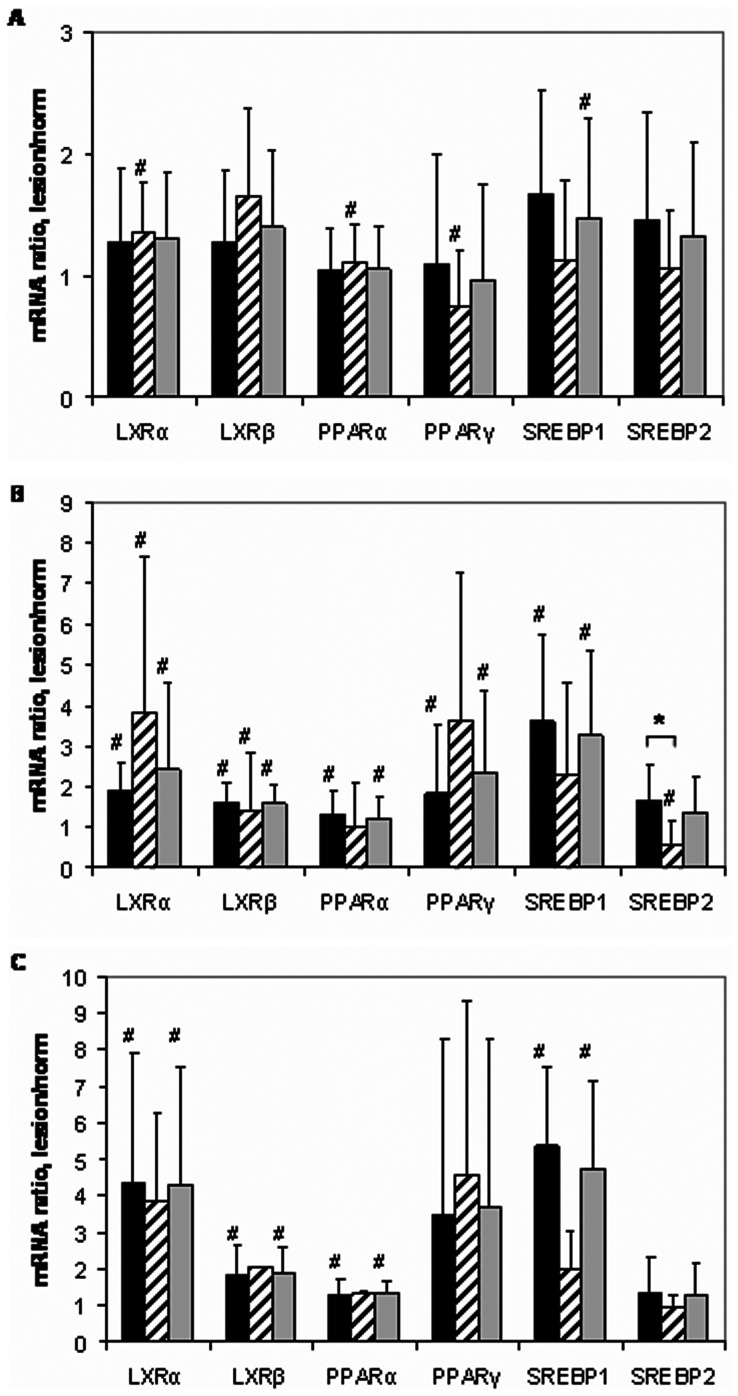
The ratios of the contents of mRNAs encoding for lipid sensors/transcriptional regulators in pairs of injured/intact aortas from men (black bars), women (hatched bars), and in combined M+F population (grey bars). Symbol # shows significant difference from 1.0. Symbol * makes a note gender difference. A, lesion of type I/intact tissue ratios (men, n = 11, women, n = 6); B, lesion of type II/intact tissue ratios (men, n = 13, women, n = 5); C, lesion of type Va/intact tissue ratios (men, n = 9, women, n = 2).

Among the group of LXR/PPAR/SREBP mRNAs, the contents of PPARγ and SREBP1 mRNAs showed significant gender differences with higher levels in intact male or female aortas, respectively (Fig. 1A). Sex difference in the contents of PPARγ mRNA persisted also in lesions of type I (Fig. 1B). An additional level of sex differentiation was found in lesions of type II, where the ratio of lesion/intact aorta for SREBP2 mRNA contents in women tissues was significantly below 1.0, while in men this ratio had a tendency to rise (Fig. 2). In combined male plus female population, no age correlations of LXR/PPAR/SREBP mRNA contents were found in intact aorta (n = 21) and lesions of types I (n = 17) and II (n = 18). However, in combined (n = 11) and male (n = 9) populations, LXRα mRNA correlated positively with age in lesions of type Va. Unexpectedly, only in female population, positive age correlations were found for LXRα, PPARγ, SREBP1 and SREBP2 mRNAs in lesions of type II. The later finding should be taken guardedly due to small population size (n = 5).

**Figure 3:**
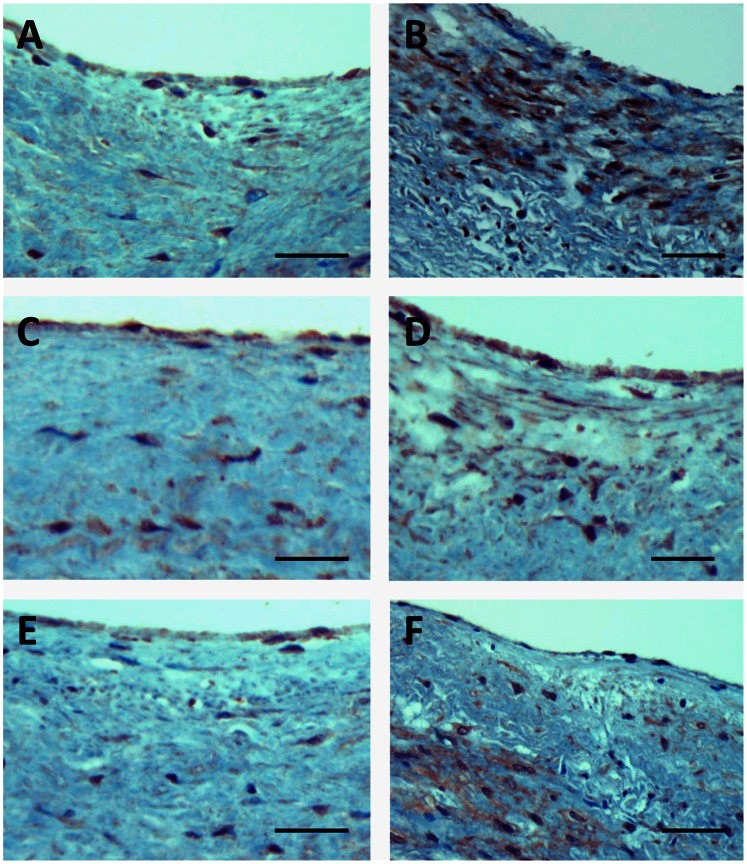
Increase in LXRα, PPARγ, and SREBP1 (B, D, F, respectively) immunopositivity in atherosclerotic lesions (fibroatheroma) compared with those (A, C, E, respectively) in non-atherosclerotic segments of the aorta. Immunohistochemistry; ABC immunoperoxidase method. Bars = 100 µm (**A**–**F**).

As shown in Figures 2A-C, the content of all but one (SREBP2) tested mRNAs encoding for lipid sensors/transcriptional regulators progressively increased during atherosclerosis progression. More prominent rise was observed for LXRα, PPARγ, and SREBP1 mRNAs as compared with LXRβ and PPARα mRNAs. The levels of SREBP2 mRNA were resistant to the disease. For markers (LXRα, PPARγ, and SREBP) which were found to demonstrate the most prominent increases in mRNAs levels, we further undertook immunohistochemical staining in order to see if would be possible to visualize such increases. Indeed, immunohistochemistry unambiguously demonstrated increases in LXRα, PPARγ, and SREBP1 immunopositivity levels in atherosclerotic lesions compared with non-atherosclerotic segments of the aorta (Fig. 3A-F).

### Correlations between mRNAs in Intact and Injured Aorta

**Figure 4:**
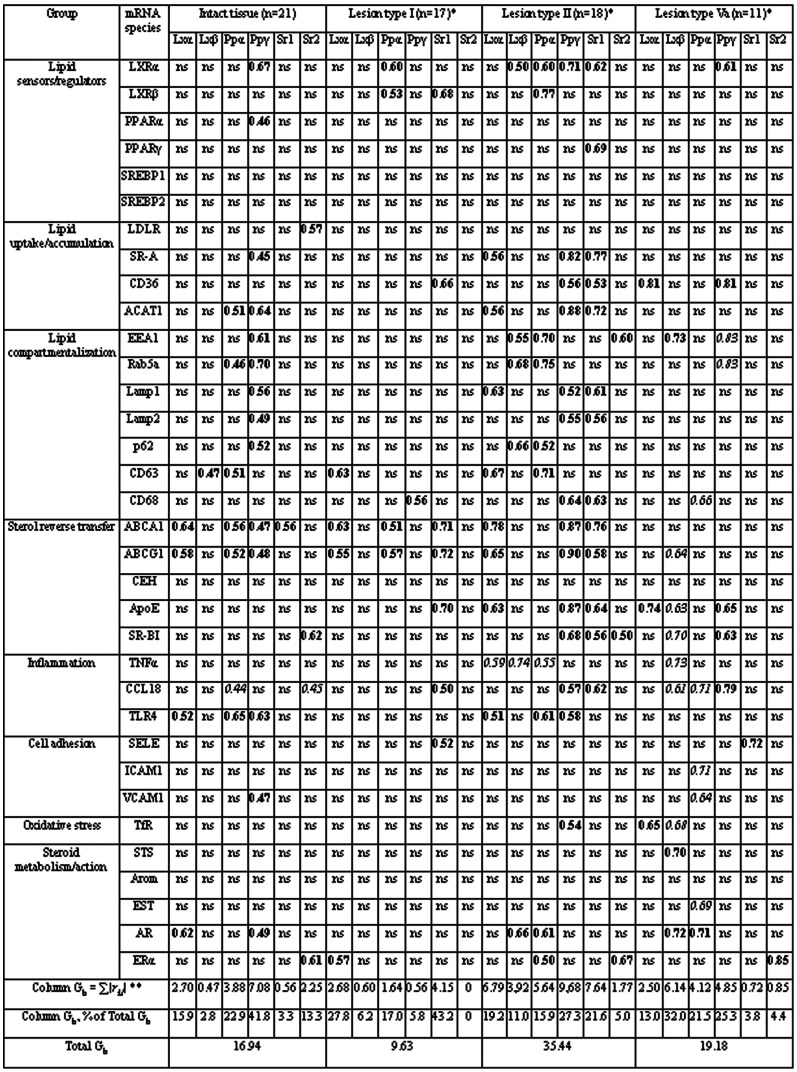
Correlations between the contents of mRNAs coding for lipid sensors/transcriptional regulators and mRNA species of other functional groups in intact and injured human aortas. Significant Rs values for positive and negative correlations are shown in bold and italics, respectively. * - Type I lesion -initial lesion; type II lesion - fatty streak; and type Va lesion – fibroatheroma [14], [15]. ** - *r_kl_* values within LXR/PPAR/SREBP group were halved, and each half was ascribed to the correspondent column (e.g. Rs of 0.60 for correlation LXRα-PPARα in lesion of type I gives additional 0.30 to the column for LXRα while the same value of 0.30 was subtracted from the sum of PPARα column. *Abbreviations:* ABCG1, ATP-binding cassette transporter G1; AR, androgen receptor; SELE, E-selectin; Arom, aromatase; EST, estrogen sulfotransferase; Lamp 1/2, lysosome-associated membrane glycoproteins 1 and 2; LDLR, low density lipoprotein receptor; STS, steroid sulfatase; TNFα, tumor necrosis factor α; TfR1, transferrin receptor 1; VCAM1, vascular cell adhesion molecule 1 (other abbreviations are given in the text earlier).

Figure 4 demonstrates the presence of multiple correlations between mRNAs encoding for lipid sensors/transcriptional regulators and other mRNA species both in intact and injured aorta. The abundance and pattern of these correlations were individual for each tissue type, although a number of them were conservative, i.e. found in at least two tissue types.

Correlations between mRNAs contents underwent to significant quantitative and qualitative changes in the course of atherogenesis. The cohesiveness of variables had a wavy shape with maximal graph weight found in lesions of type II and minimum in lesions of type I (Fig. 4).

The involvement of individual members of the group of mRNAs encoding for lipid sensors/transcriptional regulators in correlations were subjected to even more pronounced changes (Fig. 4). PPARγ mRNA was the main contributor to correlations in intact tissue. In initial lesions, a significant rise in contribution of SREBP1 mRNA was observed. In fatty streaks, the contribution of all six mRNA species to correlations was equalized. In fibrolipid plaques, SREBP1 and SREBP2 mRNAs lost near all their correlations. The main features of this lesion type were enormous amounts of negative correlations that involved LXRβ and PPARα mRNAs. An additional level of complexity in evolution of correlations arise due to change in sign of correlations PPARγ-EEA1 (early endosome antigen 1), and PPARγ-Rab5a (Ras-related small GTPase 5A) at transition from intact tissue to lesion of type Va.

With the exception of PPARγ mRNA, the contents of mRNAs for lipid sensors/transcriptional regulators in early lesions (of types I and II) correlated with their levels in intact tissue. However, in advanced lesions (of type Va), LXRα and SREBP1 mRNAs lost correlations with their contents in intact tissue (data not shown).

### Expression of mRNAs in Macrophages

**Figure 5:**
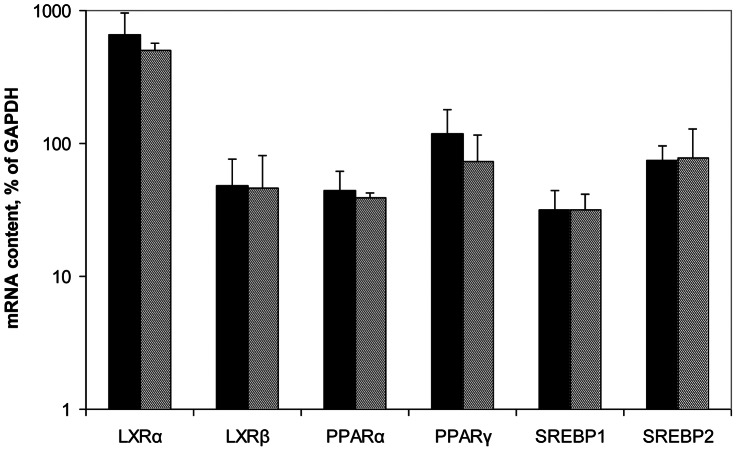
Expression levels of mRNAs encoding for lipid sensors/transcriptional regulators in control macrophages from men (n = 5, black bars) and women (n = 3, hatched bars).

In control macrophages, expression levels of all mRNAs encoding for lipid sensors/transcriptional regulators and particularly LXRα (Fig. 5) exceeded those in intact aorta (cf. with Fig. 1). No gender differences were observed.

**Figure 6:**
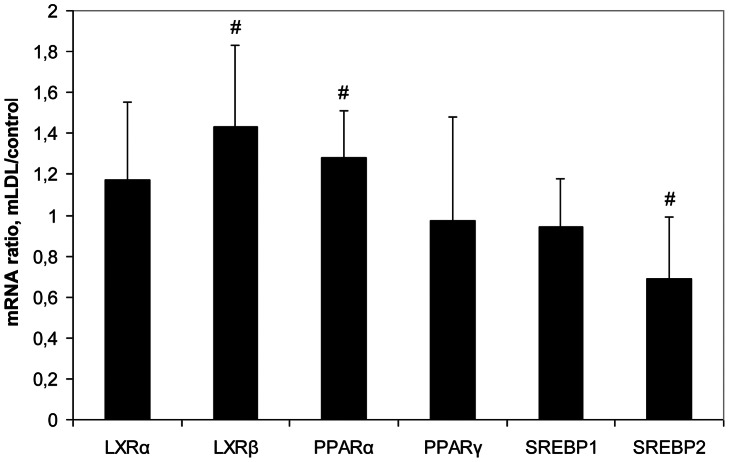
The ratios of the contents of mRNAs encoding for lipid sensors/transcriptional regulators in lipid-loaded and control macrophages (n = 8). Symbol # shows significant difference from 1.0.

The loading of macrophages with lipids from mLDL that to some extent mimics foam cells formation in atheroma was associated with significant changes in expression of 3 from 6 mRNA species: an increase in the contents of LXRβ and PPARα mRNAs and decrease in the level of SREBP2 mRNA (Fig. 6). Thus, the changes in mRNAs expression in whole aorta intima during atherosclerosis progression (Fig. 2) and in macrophages after lipid loading have only partial similarity.

**Figure 7:**
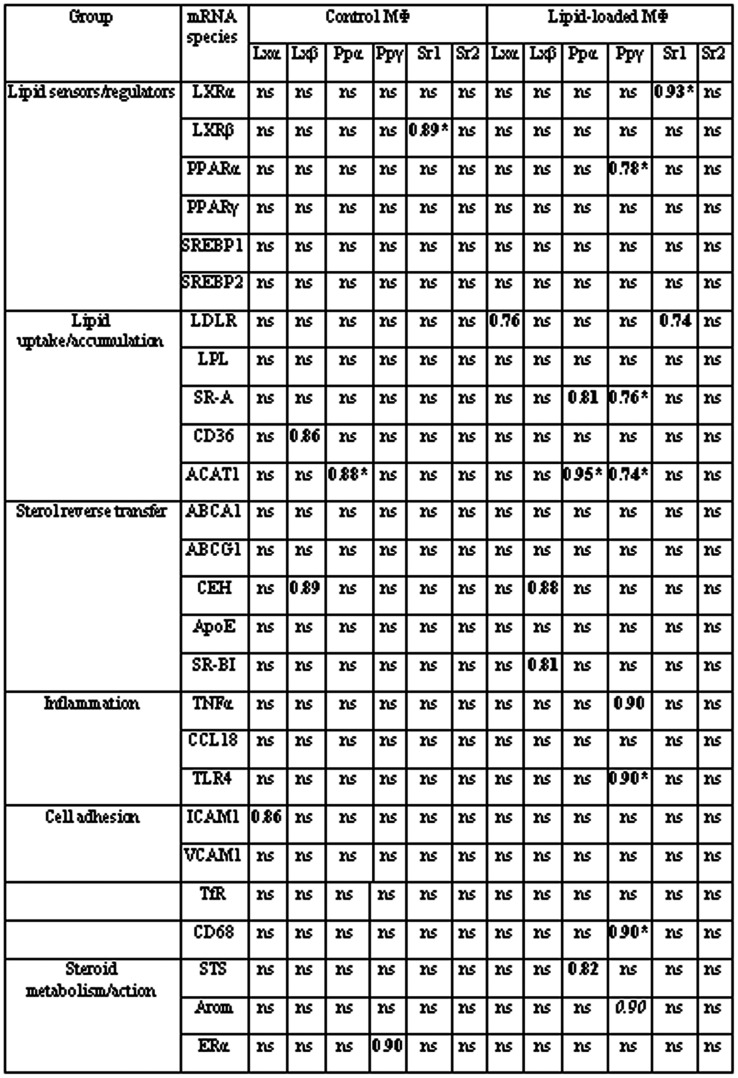
Correlations between the contents of mRNAs encoding for lipid sensors/transcriptional regulators and mRNA species of other functional groups in control and lipid-loaded macrophages. Positive and negative correlations are shown in bold and italics, respectively. Asterisks mark off correlations common for macrophages and intact and/or injured aorta. *Abbreviations:* as in Figure 4; LPL, lipoprotein lipase.

An analysis of correlations between the contents of different mRNA species in control macrophages revealed in all 6 correlations, 2 of which coinciding with correlations in aorta (Fig. 7). Lipid loading of macrophages was associated with 2.5-fold rise in the number of correlations with significant increase in shared for macrophages and aorta correlations. Interestingly, correlation LXRβ-CEH mRNA found in both control and lipid-loaded macrophages was absent in any type of aorta tissue.

## Discussion

First of all, correlations between mRNAs are considered herein as a reflection of relationships between mRNA species in the majority of cells that compose the analyzed tissue. Thus, the shifts in both cellular composition and inner links between mRNAs can be responsible for the observed changes in correlations during pathogenesis. The interpretation of correlations is further complicated by the necessity of taking into account the availability of LXR and PPAR ligands that control activities of these transcription factors.

According to the theory of correlation adaptometry [20], [21], unfavorable conditions induce more tight coupling between analyzed parameters in different biological systems. For example, the cohesiveness between biochemical parameters rises with disorder severity and drops after successful treatment. From this point of view, the most stressing conditions for intima cells exist in fatty streaks, since both amount of correlations and correlation graph weight are maximal in this lesion among aorta tissue types studied (Fig. 4). Unexpectedly, initial lesions are characterized by the lowest level of involvement of LXR/PPAR/SREBP mRNA group in correlations. This discrepancy might be attributed to interference of mRNA from macrophage-derived foam cells with mRNA from resident intima cells. However, no one from several correlations shared for intact intima and lipid-loaded macrophages (PPARα-PPARγ, PPARγ-SR-A, PPARα- acyl-CoA-cholesterol acyltransferase 1(ACAT1), PPARγ-ACAT1, PPARγ- Toll-like receptor 4(TLR4)) was preserved in initial lesions. Accordingly, some more fundamental factors responsible for lowering of LXR/PPAR/SREBP group involvement in coupling of gene expression in initial lesions can be suggested. Taking into account that initial lesions are found even in infants, foam cells formation in these lesions might perform an adaptive function by reduction in modified LDL concentrations in extracellular space of intima. This reduction will result in decrease of availability of LXR and PPAR ligands for resident cells and, consequently, in diminishing of transcriptional activities of these nuclear receptors (which include induction of SREBP expression as well). On the contrary, massive formation of foam cells (including smooth muscle-derived) in fatty streaks is accompanied by reactive oxygen species production which favors LXR and PPAR ligands accumulation and, in the end, participation of LXR and PPAR in gene regulation. The drop in abundance of correlations in fibrolipid plaques as compared to fatty streaks may relate to qualitative changes in regulation of LXR and PPAR transcriptional activities. Significant rise in proportion of negative correlations with LXRβ and PPARα mRNAs is a hallmark of this advanced lesion. Several mechanisms responsible for transrepressor activities of LXR and PPAR including their phosphorylation and SUMOylation have been described [22], [23]. One can speculate that, in fibrolipid plaques, simultaneous presence of both forms of LXRβ and PPARα, activating and inhibitory, leads to quenching of a part of correlations depending on the context of target regulatory sequences. Notably, a number of of PPARγ mRNA positive correlations found in fatty streaks persist in fibrolipid plaques. On the other hand, positive correlations of PPARγ with EEA1 and Rab5 mRNAs in intact aorta transform into negative ones in atheromas.

Correlations between mRNAs can indicate the following: the influence of one gene (through its protein product) on the expression of the other; mutual influences; and, finally, dependence of both on shared regulator(s). Regulatory regions of a multitude of genes involved in lipid turnover and inflammation have been shown to contain response elements that render sensitivity to LXR/PPAR/SREBP group of transcription factors. However, many of controlled factors within lipid turnover and inflammation groups have a capability in turn, directly or indirectly, to affect expression of members of LXR/PPAR/SREBP group. For example, it was shown that CD36 in ligand-dependent manner can use signaling machinery of Toll-like receptors [24], which is known to inhibit LXR-dependent transcription including *ATP-binding cassette transporter A1 (ABCA1)* gene [25]. Furthermore, mutual dependence of expression within LXR/PPAR/SREBP group (reviewed in [7]) potentially can result in correlations that reflect activity of shared regulator(s). Thus, the significance of correlations should not be overestimated in terms of mechanistic interpretation. Nevertheless, shared correlations can point to similarities in regulatory mechanisms of expression. It is of particular value for genes whose regulation is still obscure (e.g. C–C motif-containing chemokine 18 (CCL18) or endosome/lysosome components).

Atherosclerosis is known to be a gender-dependent disease (reviewed, e.g., in [26]). The predominance of SREBP1 mRNA in female aortas and PPARγ mRNA in male aortas found in this study (Fig. 1A,B) may relate to this issue. Moreover, the correlation between SREBP2 and estrogen receptor α (ERα) mRNAs was observed among the most conservative correlations (Fig. 4). Together with gender difference in changes of the contents of SREBP2 mRNA in fatty streak (Fig. 2B), the data allow speculate that atheroprotective effects of estrogens may involve induction of SREBP genes.

The analysis of correlations between the contents of LXR/PPAR/SREBP mRNAs in intact and injured tissue was intended for elucidation of a role of systemic factors in regulation of these mRNAs during the disease progression. Unlike other mRNA species, PPARγ mRNA in any lesion type did not correlate with its content in intact tissue. It may mean that systemic factors have only limited if any influence on the expression of PPARγ in injured tissue. The loss of coupling between the contents of LXRα and SREBP1 mRNAs in advanced lesion and intact tissue may relate to changes in cell composition though a shift in predominant regulators cannot be ruled out as well.

The response of macrophages to lipid loading (up-regulation of LXRβ and PPARα mRNAs) only partially coincides with changes in LXR/PPAR/SREBP mRNAs group in the aortic intima during atherogenesis. It may mean that the regulatory mechanisms in macrophages and resident cells in the aorta differ. However, similarly to fatty streak, lipid loading induces significant rise in the number of mRNAs correlations in macrophages. It points to “stressful” influence of lipids on both resident cells of the arterial wall and macrophages, although the ways of this influence can be cell-specific. For example, correlation between LXRβ and SR-BI mRNAs in lesion of type Va was positive while in macrophages this correlation had an opposite sign.

In conclusion, transcriptional regulators of lipid metabolism in the aorta are themselvesthe objects of the regulation at the level of transcription. As a result of atherosclerosis progression, the contents of mRNAs for LXRα/β, PPARα/γ, and SREBP1 progressively rise. However, the coupling between mRNAs for LXR/PPAR/SREBP and other mRNA species changes in the course of atherogenesis in non-linear manner and individually for each type of mRNA. These findings should be taken into consideration when targeting LXR/PPAR/SREBP therapy is contemplated.
